# Efficient-Lightweight YOLO: Improving Small Object Detection in YOLO for Aerial Images

**DOI:** 10.3390/s23146423

**Published:** 2023-07-15

**Authors:** Mengzi Hu, Ziyang Li, Jiong Yu, Xueqiang Wan, Haotian Tan, Zeyu Lin

**Affiliations:** 1 School of Software, Xinjiang University, Urumqi 830091, China; hmz123@stu.xju.edu.cn (M.H.); yujiong@xju.edu.cn (J.Y.); wanxueqiang@stu.xju.edu.cn (X.W.); 107552204777@stu.xju.edu.cn (Z.L.); 2 College of Information Science and Engineering, Xinjiang University, Urumqi 830046, China; tanhaotian@stu.xju.edu.cn

**Keywords:** aerial images, small-object detection, model architecture, SPP, loss function

## Abstract

The most significant technical challenges of current aerial image object-detection tasks are the extremely low accuracy for detecting small objects that are densely distributed within a scene and the lack of semantic information. Moreover, existing detectors with large parameter scales are unsuitable for aerial image object-detection scenarios oriented toward low-end GPUs. To address this technical challenge, we propose efficient-lightweight You Only Look Once (EL-YOLO), an innovative model that overcomes the limitations of existing detectors and low-end GPU orientation. EL-YOLO surpasses the baseline models in three key areas. Firstly, we design and scrutinize three model architectures to intensify the model’s focus on small objects and identify the most effective network structure. Secondly, we design efficient spatial pyramid pooling (ESPP) to augment the representation of small-object features in aerial images. Lastly, we introduce the alpha-complete intersection over union (α-CIoU) loss function to tackle the imbalance between positive and negative samples in aerial images. Our proposed EL-YOLO method demonstrates a strong generalization and robustness for the small-object detection problem in aerial images. The experimental results show that, with the model parameters maintained below 10 M while the input image size was unified at 640 × 640 pixels, the *AP_S_* of the EL-YOLOv5 reached 10.8% and 10.7% and enhanced the *APs* by 1.9% and 2.2% compared to YOLOv5 on two challenging aerial image datasets, DIOR and VisDrone, respectively.

## 1. Introduction

In recent years, aerial image object detection has been widely used due to the rapid development of unmanned aerial vehicles (UAV) and satellite remote-sensing technology. As a branch of object detection, aerial image object detection can not only be applied in areas of defense, such as military monitoring, missile guidance, and UAV combat systems, but also plays an important role in our day-to-day lives, such as through environmental management, traffic monitoring, and urban planning. Therefore, aerial image object detection is of great research value and significance [1].

Traditional methods for object detection rely on manually designed features, which are inefficient and encounter difficulty in exploiting the relevance of massive image data. Recently, researchers have introduced deep learning techniques to the field of object detection because their advanced semantic features and learning capabilities can provide a powerful technical framework for extracting the rich feature information contained in high-resolution aerial images. Meanwhile, with the development of deep learning techniques, in addition to the commonly used convolution neural network (CNN) [2], recurrent neural network (RNN) [3], AutoEncoder (AE) [4], and generative neural network (GAN) [5] methods have been widely used in object detection. In addition, the emergence of challenging natural image datasets, such as the PASCAL Visual Object Classes (PASCAL VOC) [6,7] and Microsoft Common Objects in Context (MS COCO) [8], has further advanced the development of object detection. Accordingly, increasing numbers of object-detection algorithms with excellent performance on natural images have emerged, with representative algorithms including Faster Regions with CNN (Faster R-CNN) [9], RetinaNet [10], and the You Only Look Once (YOLO) series [11,12,13,14,15,16,17]. However, aerial and natural images differ significantly, mainly in terms of the following: Firstly, the number of objects in aerial images is considerably higher than in natural images. Secondly, the distribution of objects in aerial images is denser than in natural images. Thirdly, aerial images present significant scale variation for similar objects due to the view angle, and most targets are small. Fourthly, aerial images are often high-resolution.

The aerial image object-detection task has strict requirements for the detection model. Firstly, the object-detection model needs to meet real-time processing. Secondly, the object-detection model needs to consider the model’s parameters. Embedded platforms such as UAVs and airborne processors have limited computational resources; thus, ensuring that real-time detection models are installed on these platforms is also a significant challenge. Currently, in areas such as UAV remote sensing and space exploration, the chips that are used to implement image-processing techniques such as object detection usually require a model size of less than 10 MB to be loaded. For example, field programmable gate array (FPGA) chips tend to be used for small volumes, customization, and real-time demanding applications [18]. For inference, a sufficiently small model can be stored directly on the FGPA without the limitation of memory bandwidth. In this paper, we propose a real-time object detector, which we want to support embedded platforms such as UAVs and airborne processors.

Consequently, the detection performance of object-detection algorithms, which are effective for natural scenes, does not meet the needs of practical applications when implemented directly on aerial images. Two main technical challenges exist. Firstly, aerial image datasets contain many small objects, and small targets usually lack sufficient appearance information, resulting in high false and missed detection rates for small objects in detection tasks. Secondly, practical aerial image object-detection tasks necessitate the consideration of computational costs, as well as the real-time processing of images, and thus present requirements regarding the number of parameters and the topicality of the model.

To address the above technical challenges, we have focused on improving the detection performance of lightweight frameworks for small objects in aerial images. The YOLO family of detectors are one-stage object detection models that can directly predict both the position and class of objects from an input image, meeting the requirements of real-time image processing. According to our literature review, You Only Look Once version 7 (YOLOv7) [17] is the latest version of YOLO and provides the best performance for object-detection tasks in natural scenes. However, YOLOv7 suffers from a severe problem with aerial images, namely that YOLOv7 is preoccupied with accuracy and uses too many tricks, thus consuming too many computational resources and failing to meet the requirements of a lightweight model. Therefore, considering the complexity and accuracy of the model, we have chosen the S-scale You Only Look Once version 5 (YOLOv5s) algorithm [15] as the baseline model for this paper.

Due to the difference between natural and aerial images, YOLOv5s fails at the task of detecting objects in aerial images. We enhanced the original YOLOv5s model in three ways. Firstly, YOLOv5s includes a continuous downsampling operation between the backbone and neck, resulting in instances where small objects that cover less feature information may directly lose their information. To solve this problem, we changed the connection of feature maps to indirectly control the ratio of both low-level and deep feature maps in the model architecture and maximize the retention of small-object feature information, making the model better adaptable to small objects. Then, spatial pyramid pooling (SPP) [19] was applied to YOLOv5s to enhance the information fusion of local and global features, but this was not effective in practical small-object detection tasks. Thus, the ESPP approach was designed to replace SPP and more effectively retain the detailed information of small objects. Thirdly, the complete-intersection over union (CIoU) [20] of YOLOv5s was replaced by the α-CIoU loss function [21], which enabled the model to obtain higher-quality anchor frames. Based on the above, we proposed the efficient-lightweight You Only Look Once version 5 (EL-YOLOv5), which successfully balanced accuracy and speed in object detection. The experimental results showed that EL-YOLOv5 met the requirements of embedded platform deployment and outperformed the general YOLOv5 model in terms of aerial image object-detection accuracy, while the S-scale EL-YOLOv5 (EL-YOLOv5s) also outperformed the YOLOv7 model of comparable size.

The contributions of this paper are summarized below:

1. We modified the model architecture of YOLOv5. Intending to introduce high-resolution low-level feature maps, we evaluated three model architectures through several rounds of experiments and then analyzed the reasons for their superior or inferior performance and architectural characteristics; finally, we selected the best-performing model. The top-performing model architecture managed to maximize the precision of the model in detecting small objects while imposing only a marginal increase in computational overhead.
2. We designed the ESPP method based on the human visual perception system to replace the original SPP approach, which enhanced the model’s ability to extract features from small objects.
3. We used α-CIoU to replace the original localization loss function of the object detector. The α-CIoU function could control the parameter *α* to optimize the positive and negative sample imbalance problem in the bounding box regression task, allowing the detector to locate small objects more quickly and precisely.
4. Our proposed embeddable S-scale EL-YOLOv5 model attained an *AP_S_* of 10.8% on the DIOR dataset and 10.7% on the VisDrone dataset. This is the highest accuracy achieved to date among the available lightweight models, showcasing the superior performance of our proposal.

## 2. Related Work

### 2.1. General Object Detection

Object detection synthesizes complex vision tasks such as segmentation, recognition, and detection into a unified problem. The accuracy and real-time performance of such an approach are critical benchmarks for the efficacy of a comprehensive computer vision system. In simple terms, object detection solves the problem of where and what objects are in an image. Currently, object detection based on deep learning techniques can be classified into two main types: two-stage and one-stage object-detection algorithms. The two-stage approach [9,22,23] splits the object-detection process into two steps: the task in the first step is to obtain candidate regions from the displayed region proposals. The detection task in the second step uses a detection network to achieve classification recognition and bounding box regression. The two-stage object detection models are highly accurate, but the detection speed is often limited accordingly. Representative models include Faster R-CNN, mask regions with CNN (Mask R-CNN) [22], and cascade regions with CNN features (Cascade R-CNN) [23].

Unlike two-stage object detection algorithms, one-stage methods [10,11,12,13,14,15,16,17,24] discard region proposals and directly use bounding box regression for object classification and localization. One-stage object detection models significantly improve detection efficiency and reduce computational overhead. However, one-stage methods suffer from a class imbalance, making two-stage methods superior in terms of detection accuracy. As technology continues to evolve, one-stage object detection methods are constantly being upgraded, and their detection accuracy is improving accordingly. Representative models include the single shot multibox detector (SSD) [24], RetinaNet, and the YOLO series.

YOLO [11], the first model in the YOLO series of one-stage object detection algorithms, aimed at an extremely low runtime. You Only Look Once version 2 (YOLOv2) [12] used Darknet-19 as the new feature-extraction network while borrowing ideas from the region proposal network (RPN) [9] proposed by Faster R-CNN and introducing prior anchors based on the previous YOLO model. However, YOLOv2 still had a low detection accuracy for dense objects. To further improve the detection accuracy of the model, You Only Look Once version 3 (YOLOv3) [13] introduced residual connections from the deep residual network (ResNet) [25], updated the backbone network from Darknet-19 to Darknet-53, and borrowed the idea of feature pyramid networks (FPN) [26] to construct three different scales of feature maps. You Only Look Once version 4 (YOLOv4) [14] was further optimized from YOLOv3 by incorporating the CSPDarknet53 architecture for the backbone and an FPN for the neck, combined with path aggregation network (PAN) architecture [27], to enhance information fusion. Meanwhile, YOLOv4 used mosaic data augmentation and introduced the Mish activation function and CIoU loss function. YOLOv5 further extended the cross-stage partial network structure [28] of the YOLOv4 backbone network to the neck and proposed the spatial pyramid pooling–fast (SPPF) module. You Only Look Once version 6 (YOLOv6) [16] introduced the RepVGG structure [29] into the YOLO model architecture to enhance the adaptability of the model to GPU devices. The YOLOv7 detection algorithm is similar to YOLOv5 and was mainly optimized for model structure re-parameterization and dynamic label assignment problems.

In addition to the continuous optimization of the mainstream YOLO model, certain excellent algorithms have emerged and optimized the YOLO model in special domains [30,31,32,33]. The YOLOv3—four-scale detection layers (YOLOv3-FDL) [32] significantly improved the ability of YOLOv3 to detect GPR images with a high missed detection rate of small cracks feature, mainly through multiscale fusion structures, advanced loss function, and hyperparameter optimization. Jiawen Wu et al. [33] proposed a local adaptive illumination-drive input-level fusion (LAIIFusion) module, which can effectively sense the illumination in different scenes and enable realistic remote-sensing image object-detection tasks to adapt to changing lighting conditions. These excellent algorithms demonstrated that YOLO is an object-detection algorithm with a relatively wide application area and an excellent performance.

It has been found that the detection accuracy of the YOLO algorithms improved significantly through these continuous improvements, but with this comes the burden of computational overhead and model size, which is detrimental to the establishment of the model in a particular application domain. Among the current YOLO families, YOLOv5 not only provides an effective balance between speed and model complexity for object detection but also offers the easiest deployment. Therefore, considering the needs of the application domain of aerial image object detection, we have chosen YOLOv5s as the baseline framework for this study.

### 2.2. Aerial Image Objects Detection

The most prominent feature of aerial images is the high image resolution. Nevertheless, small objects in aerial images still have a low resolution, often comprising tens or even just a few pixels, making the learning of small-object feature information difficult for the model. Object-detection algorithms designed for natural scenes do not perform well on high-resolution images containing a dense distribution of small targets. Many researchers have adopted different schemes to address this problem.

DBNet [34] was based on the Cascade R-CNN to train the detector and used ResNext-101 [35] as the backbone network to incorporate deformable convolutions and enhance the network’s ability to handle multiscale objects in aerial images. The drone networks with effective fusion strategy (DNEFS) project [34] used YOLOv5, Cascade R-CNN, and FPN as the baseline models while incorporating attention mechanisms, double-headers, and other effective strategies to achieve a higher detection accuracy. Transformer prediction heads–You Only Look Once version 5 (TPH-YOLOv5) [36] addressed the problem of scale variation in aerial image overhead angles using transformer prediction heads (TPH), the convolutional block attention model (CBAM) [37], and a series of data-enhancement strategies based on the YOLOv5 model. The stronger visual information for tiny-object detection (VistrongerDet) project [38] integrated the FPN, region of interest (ROI), and head-level data enhancement components to largely mitigate the detrimental effects of aerial image scale variation and small object size on detection.

An analysis of the above well-performing algorithms revealed two problems with the current aerial image object-detection algorithms that address the low detection accuracy for small objects. First, most of these algorithms focused on the average detection accuracy of the model for aerial images, rather than for small objects. Second, these algorithms tended to consider only the use of cascaded networks and the addition of new feature enhancement modules to improve model accuracy, ignoring model complexity and memory loss. At the same time, most of these algorithms were adapted from two-stage object detection models, which have a high detection accuracy but incur substantial resource overheads in the computation process, leading to their low application value. In contrast to existing research, the present study fully considered the computational overhead and addressed the problem of low detection precision for densely distributed small objects in aerial image object-detection tasks.

## 3. Materials and Methods

### 3.1. Fundamental Models

#### 3.1.1. Baseline Model

The YOLO family has become a very popular model framework in the field of object detection in recent years. Compared with the existing YOLO models, YOLOv5 provides a good balance between memory loss and model accuracy. The structure of YOLOv5 can be broadly subdivided into three parts according to function: the backbone network, neck, and prediction head. Firstly, the backbone network mainly extracts features from the input images to pave the way for subsequent object recognition and localization. Secondly, the neck further enhances the fusion of the feature information extracted by the backbone and constructs three scales of feature maps. Finally, the head achieves object classification and localization based on these feature maps and completes the object detection task.

In conclusion, as a one-stage object detection algorithm, YOLOv5 has been widely applied in various fields due to its simple network structure and high detection efficiency. YOLOv5 can control the size and complexity of the model by setting different width and depth coefficients, which can in turn be divided into four scales: S, M, L, and X. The network architectures of these different scales are identical. Considering that our model may need to be deployed in the application domain, we had to limit the computational resource overhead to a certain extent. We selected YOLOv5s, which is of relatively small size and low complexity, as the baseline model. The YOLOv5s balances detection precision and speed, meeting the requirements of a lightweight embedded model.

#### 3.1.2. Receptive Field Block

The receptive field block (RFB) [39] is a module that enhances the feature representation of neural networks by mimicking the receptive field mechanism of the human visual system. The RFB further models the relationship between receptive field size and receptive field eccentricity in the structure of the human visual system by constructing multibranch convolutional layers with different sizes of convolutional kernels and atrous pooling or convolution layers. With this structure, on the one hand, the RFB can improve the feature representation capability of lightweight models with a lower computational burden. On the other hand, the RFB module can improve the discriminative power and robustness of the object features, improving the performance of object detectors. The RFB has proven to be an effective method when successfully applied to improve one-stage object detectors.

### 3.2. Proposed Model

The original YOLOv5s exhibited a suboptimal performance in practical aerial image object detection tasks, especially for small objects. To counter the shortcomings inherent to the existing model and address the unique challenges posed by aerial image detection tasks, we enhanced the model in three key areas: the model architecture, the SPP module, and the loss function.

**Figure 1:**
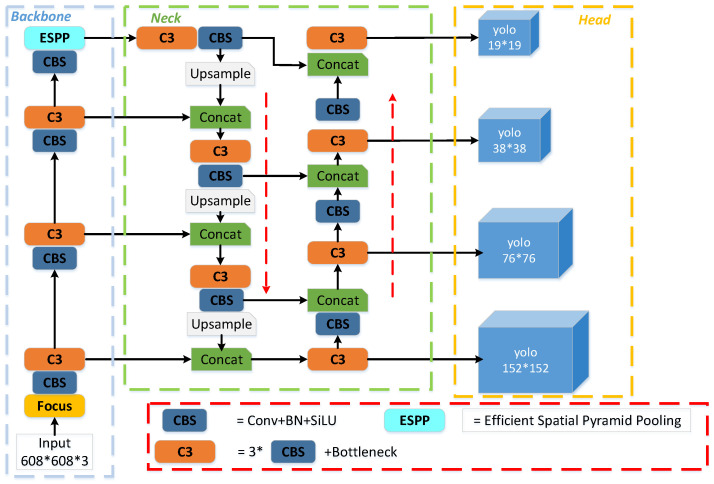
The structure of EL-YOLOv5. In contrast to the baseline model, the 152 × 152 output layer indicates the addition of the remaining connected small-object detection head. Meanwhile, we designed a new ESPP module to improve performance.

The architectural blueprint of the proposed model, EL-YOLOv5, specifically tailored to the domain of aerial image object detection, is depicted in Figure 1.

#### 3.2.1. Model Architecture

According to the YOLOv5s model architecture, the process of object detection comprises three main parts: extracting features; building feature maps with different scales; and regressing feature maps for classification and regression. Accordingly, the feature map is of great importance for object detection. We can further classify feature maps into low-level and deep feature maps according to their distance from the input layer.

**Figure 2:**
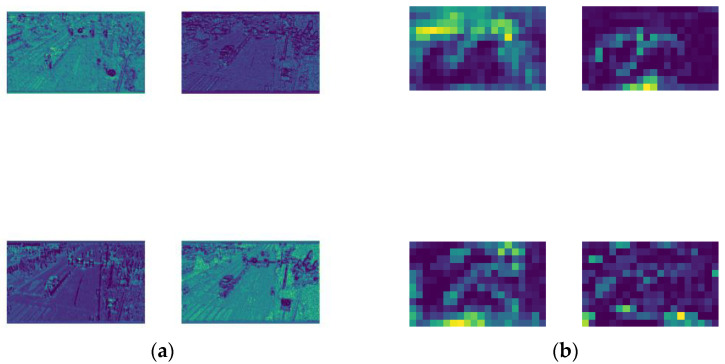
Example visualization results. (**a**) Example visualization results of low-level feature maps; (**b**) example visualization results of deep feature maps.

Low-level feature maps are located close to the input layer and are extracted by the shallow neural web of the model. The shallow web has a smaller receptive field, and the overlapping area of the receptive field is also smaller. As shown in Figure 2a, low-level feature maps contain more pixel-wise information, and this fine-grained information includes the color, texture, edge, and corner information of the image. Generally speaking, low-level feature maps have a higher resolution and contain more location and detailed information beneficial for small-object detection. However, due to the smaller number of convolutions undergone, their semanticity is lower. On the contrary, deep feature maps are generally farther away from the input and closer to the output. As the image information is continuously being compressed by convolution, the receptive field of the deep web increases, as well as the overlapping region between the receptive fields. As shown in Figure 2b, deep feature maps contain more semantic information but have a lower resolution, appearing only as colored spots, and their small-object perception is poorer. Therefore, coordinating shallow and deep feature maps by improving the model architecture to increase the detection accuracy for small objects in aerial images is an urgent issue.

The backbone of YOLOv5s contains continuously downsampled convolutional layers, which have a detrimental effect on the detection accuracy of small objects. On the one hand, during the feature-extraction stage, successive downsampling continuously reduces the size of the output feature map. When the convolutional downsampling rate is too large, it may cause the small objects to be much smaller than the downsampling step size, which can easily lead to a loss of small-object feature information in the feature-extraction phase. On the other hand, YOLOv5s selects three deep feature maps for detection in the prediction stage. However, the further down the feature map is passed, the less information about small objects is retained, until no feature information about small objects is retained at all. For the above two reasons, the original YOLOv5s model performs poorly in aerial image small-object detection.

**Figure 3:**
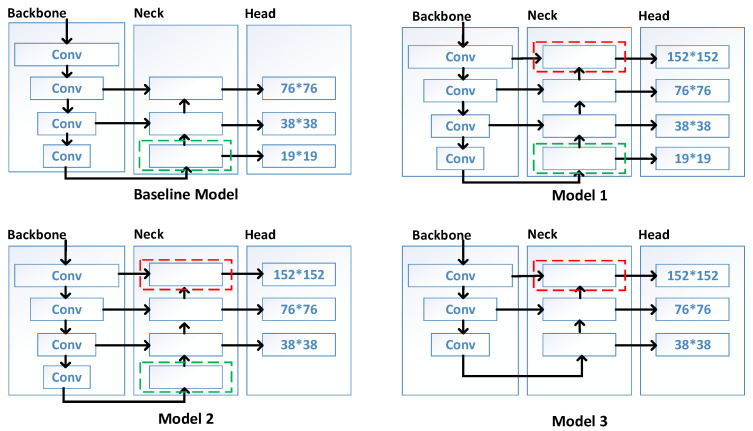
The network architecture of the baseline model and three modified model architectures. The red box represents the shallow feature level, and the green box represents the deep feature level.

To address the above issues, we aimed to introduce a low-level feature map containing more location information and other details, which would be very beneficial for small-object detection. As shown in Figure 3, we continuously adjusted the weights of the low-level and deep feature maps in the model based on the baseline model to increase the sensitivity for small-object detection as much as possible. In other words, by continuously increasing the weights of the low-level features, we made the model pay more attention to the feature maps of small objects, which maximized the capacity for small-object detection.

Therefore, we aimed to introduce a high-resolution low-level feature map and designed three model architectures based on the baseline model. Figure 3 shows the following: Model 1 retained the deep feature map and large-object detection head with a detection layer scale of 19 × 19 of YOLOv5s and introduced a low-level feature map and small-object detection head with a detection layer scale of 152 × 152 on the basic of the baseline model. Model 2 retained the deep feature map, low-level feature map, and small-object detection head of Model 1 and removed the large-object detection head of the baseline model. Model 3 retained the low-level feature map and small-object detection head from Model 1 while removing the deep feature map and large-object detection head. The proportion of shallow feature maps in the overall model architecture increased gradually from the baseline model to Model 3.

We indirectly controlled the proportion of low-level and deep feature maps in the model architecture by changing the connectivity of the feature maps. Then, we compared the experimental results of the model structure with different feature map proportions to obtain the best model architecture for small-object detection. It is thereby demonstrated that Model 1 effectively balanced both detailed and semantical information, thus avoiding the loss of fine-grained data that would otherwise hinder the detection of small objects during the continuous downsampling process observed in the baseline model. Simultaneously, by retaining the deep feature map, Model 1 maintained the capacity to control the model’s complexity while also somewhat reducing the noise accumulation resulting from introducing the low-level feature map. Ultimately, Model 1 substantially augmented the detection accuracy of YOLOv5s for small objects in aerial images while maintaining a small memory footprint.

#### 3.2.2. Efficient Spatial Pyramid Pooling

The SPP module in YOLOv5s fails to be optimally effective in aerial image small-object detection, for the following two reasons. First, the main reason for the low accuracy of small-object detection is the lack of sufficient feature information regarding the small objects themselves. Second, YOLOv5s uses the SPP module to extract information from different receptive fields, but this module does not fully reflect the semantic relationship between global and local information. Therefore, the SPP module’s ability to aggregate multiscale contextual information is inadequate, which makes it difficult for YOLOv5s to recognize objects with large-scale variability. To solve these problems, we needed to build a completely new feature fusion module to effectively integrate multiscale object features and help the model to capture more abundant and complex features without incurring too large a computational burden.

Current research shows that one can obtain excellent high-level features by increasing the depth of a model, which results in significant performance gains. However, this involves correspondingly higher computational costs, substantially slowing down the model inference.

**Figure 4:**
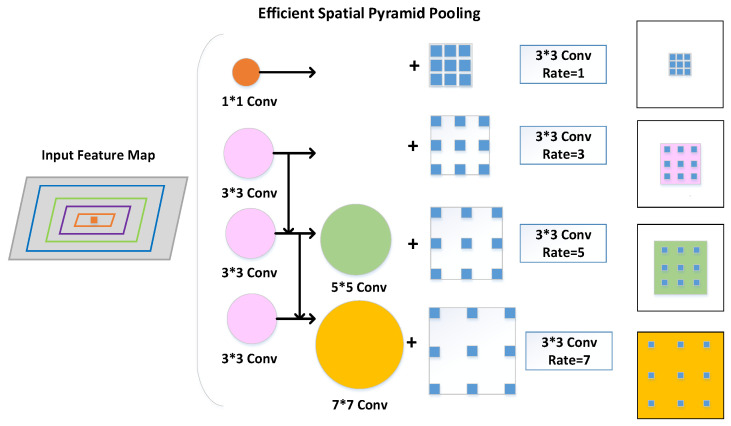
The ESPP module was constructed by combining multiple branches with different convolution kernel sizes and atrous convolution layers. The multiple kernels resemble receptive fields of different sizes, while the atrous convolution layers assign a separate atrous rate to each branch to simulate the relationship between receptive field size and eccentricity.

The paper introducing the RFB module postulated that in the human visual system, the size of the population receptive field is proportional to the eccentricity of the receptive field. By constructing a corresponding structure to model the relationship between the receptive field and the eccentricity of the model, we could enhance the feature representation capability of the model’s low-level network. Therefore, inspired by the RFB, we constructed ESPP by taking the complex backgrounds and large-scale variation in aerial images into account, as shown in Figure 4.

The implementation of ESPP is illustrated in Algorithm 1. Steps 1 and 2 of Algorithm 1 are aimed at obtaining the output channel number *C_out_*_1_ of the following ordinary convolution by parameter a, which can effectively control the size of the module. Step 3 performs an ordinary 1 × 1 convolution on the input to obtain *out_1_*. Steps 4, 6, and 8 construct the perceptual fields of 3 × 3, 5 × 5, and 7 × 7 convolutions, respectively, to obtain *out*_2_, *out*_3_, and *out*_4_ through a series of ordinary 3 × 3 convolutions. Steps 9, 10, 11, and 12 perform a 3 × 3 atrous convolution operation for *out*_1_, *out*_2_, *out*_3_, and *out*_4_, respectively, and set corresponding atrous rates of 1, 3, 5, and 7. Thus, the dependence of the receptive field on the eccentricity can be efficiently simulated and then obtained for *out*_5_, *out*_6_, *out*_7_, and *out*_8_. Step 14 concatenates the branches of different receptive fields in the channel dimension to obtain *out*_9_. Steps 16 and 17 perform a cross-scale fusion of previous outputs and the shortcut. Step 19 adds nonlinearity to the output by the rectified linear unit (ReLU) activation function. Step 20 returns the final output. It is demonstrated that Algorithm 1 can efficiently deepen the shallow network feature representation of the model to obtain more boundary information on small objects and improve the small-object detection accuracy.
**Algorithm 1 Efficient Spatial Pyramid Pooling (ESPP)****Input**: The input feature layer, *x*; The number of input channels of *x*, *C_in_*; The number of output channels of *x*, *C_out_*; The parameter that control the size of the model, *a*.**Output**: The output feature layer, *out*.  1: Control the number of output feature channels of the convolution process by *a*.
  2: *C_out1_* = *C_out_* / *a*. // In general, the parameter *a* is set to 4 or 8, which gives excellent control over the number of parameters of the model.
  3: Make an ordinary 1*1 convolution at *x*, get the output *out_1_*.
  4: Make an ordinary 3*3 convolution at *x*, get the output *out_2_*.
  5: The stacking of two 3*3 convolutions gives the same perceptual field as one 5*5 convolution:
  6: Make an ordinary 3*3 convolution at *out_2_*, get the output *out_3_*.
  7: The stacking of three 3*3 convolutions gives the same perceptual field as one 7*7 convolution:
  8: Make an ordinary 3*3 convolution at *out_3_*, get the output *out_4_*.
  9: Make a 3*3 atrous convolution with the atrous rate of 1 at *out_1_*, get the first branch output *out_5_*.
10: Make a 3*3 atrous convolution with the atrous rate of 3 at *out_2_*, get the second branch output *out_6_*.
11: Make a 3*3 atrous convolution with the atrous rate of 5 at *out_3_*, get the third branch output *out_7_*.
12: Make a 3*3 atrous convolution with the atrous rate of 7 at *out_4_*, get the third branch output *out_8_*.
13: Unify the four branch outputs into the same dimension:
14: Concat ([*out_5_*, *out_6_*, *out_7_*, *out_8_*], dimension), get *out_9_*.
15: Integrate feature information:
16: Make an ordinary 1*1 convolution at *out_1_*, get the *shortcut*.
17: *net* = out_9_*0.8 + *shortcut*.
18: Get the final output:
19: *out* = RELU (*net*). // add the non-linearity.
20: **return**
*out*.

**Figure 5:**
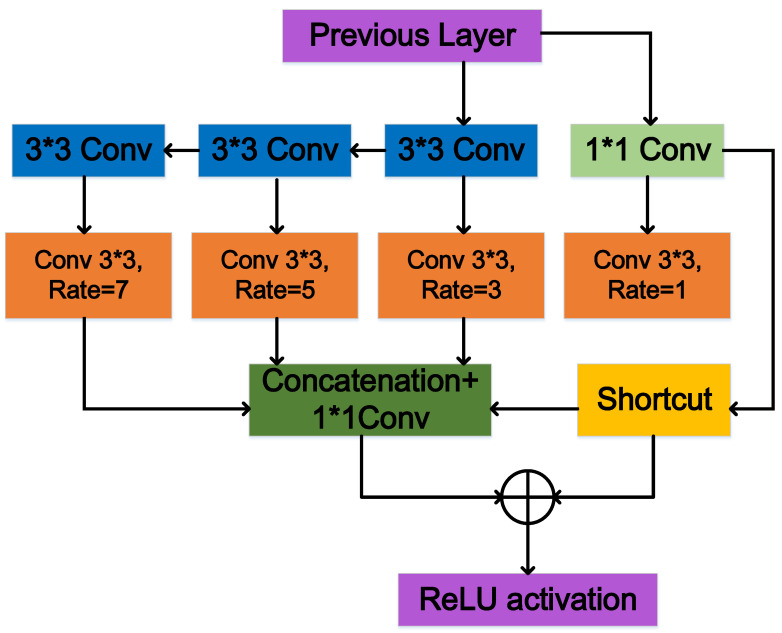
The structure of ESPP: the effect of constructing parallel convolutions of 1 × 1, 3 × 3, 5 × 5, and 7 × 7 by an ordinary convolution of 1 × 1 and a serial convolution of 3 × 3, and the effective widening of the receptive field by the superposition of the parallel convolutional layer and the atrous convolutional layer.

The structure of the ESPP method is illustrated in Figure 5. The architecture can be divided into four main branches. The first branch is a standard 1 × 1 convolution and an atrous convolution with an atrous rate of 1, which aims to maintain the original receptive fields. The second to the fourth branches consists of serial 3 × 3 convolution layers and an atrous convolution layer, aiming to quickly extract feature information from different receptive fields. ESPP could successfully simulate the relationship between the receptive field size and the eccentricity of the human visual system while making the following improvements to the detection accuracy of small objects in aerial images.

Firstly, ESPP did not use a 1 × 1 convolution layer before parallel convolution but achieved a dimensionality reduction by an intermediate parameter. This technique circumvented the issue of spatial resolution degradation in feature maps arising from superfluous convolution operations. Preventing excessive resolution loss is crucial, as it preserves detailed information on image boundaries. Secondly, we added a 3 × 3 convolution serial structure, which formed the same receptive field for 3 × 3, 5 × 5, and 7 × 7 convolutions. This operation increased the sampling rate while reducing the computational overhead, and the serial structure increased the module running speed to some extent. Thirdly, the atrous rate increased from 1, 3, or 5 to 3, 5, or 7, respectively, which further captured large-scale information, thus enhancing the detection accuracy for small objects.

In conclusion, the ESPP module designed in this paper could compensate for the deficiency in information regarding small objects. On the one hand, ESPP utilized higher-level abstract features as contexts and extracted contextual information from the pixels surrounding small objects to provide sufficiently detailed information. On the other hand, the architectural design of ESPP provided access to contextual information at multiple scales and enabled the spatial-level fusion of local and global information between objects of different scales. It is finally demonstrated the ESPP can effectively benefit the detection of small objects and improve the ability of the model to identify objects with considerable scale variations.

#### 3.2.3. Loss Function

The total loss for the object-detection task consisted of three components: the bounding box regression loss, the confidence prediction loss, and the classification loss. YOLOv5s uses the binary cross entropy loss (BCELoss) [15] to represent the confidence and classification prediction loss, while CIoU loss is employed to denote the loss for bounding box regression. The CIoU loss function considers three geometric factors, including the minimization of the normalized central point distance and the consistency of the overlap area and aspect ratio. Furthermore, CIoU loss enables the algorithm to converge quickly and present minor regression errors in different scenarios.

However, for aerial image object-detection scenarios, the existing CIoU loss function fails to obtain anchor boxes with a high regression accuracy. The accuracy of the corresponding object detection is reduced for two main reasons. On the one hand, aerial images differ from natural images and are characterized by dense object distributions and drastic scale variations. In other words, aerial images suffer from a non-uniform sample distribution. On the other hand, the CIoU loss function does not consider the problem of balancing samples that are difficult and easy to detect. To improve the accuracy of the existing detector, we introduced a novel α-CIoU loss function.

Alpha-IoU is a new family of intersection over union (IoU) loss functions [40] obtained by generalizing the power transformations using existing IoU-based losses. We began by transforming the vanilla IoU loss, which is expressed as:

(1) $$L_{IoU}=1-IoU.$$

Firstly, we performed a Box–Cox transformation [21] on *L_IoU_* to obtain the α-IoU loss function:

(2) $$L_{\alpha \text{-}IoU}=\frac{1-IoU^{\alpha }}{\alpha },\alpha >0.$$

As shown by the above equation, different forms of the IoU loss function could be obtained by controlling the power parameter *α*, such as *IoU* and *IoU^2^*. Then, we introduced a power regularization term into Equation (2) to generalize the α-IoU loss function to the following form:

(3) $$L_{\alpha \text{-}IoU}=1-IoU^{\alpha 1}+\rho^{\alpha 2}(B,B^{gt}),$$

where *α*1 > 0, *α*2 > 0, and *p^α^*^2^ (*B*, *B^gt^*) represents any regularization term calculated based on *B* and *B^gt^*. Equation (3) allows the theoretical generalization of most IoU-based loss functions according to the power parameter of *α*. Based on Equation (3), we also generalized the more complex CIoU loss function with multiple regularization terms using the same power parameter *α* to obtain α-CIoU:

(4) $$\begin{array}{l} L_{IoU}=1-IoU\Rightarrow L_{\alpha \text{-}IoU}=1-IoU^{\alpha },\\ L_{CIoU}=1-IoU+\frac{\rho^{2}(b,b^{gt})}{c^{2}}+\beta v\Rightarrow L_{\alpha \text{-}CIoU}=1-IoU^{\alpha }+\frac{\rho^{2}{}^{\alpha }(b,b^{gt})}{c^{2}{}^{\alpha }}+{\left(\beta v\right)}^{\alpha },\\ v=\frac{4}{\pi^{2}}(\arctan\frac{w^{gt}}{h^{gt}}-\arctan\frac{w}{h})2,\beta =\frac{v}{(1-IoU)+v}. \end{array}$$

By comparing *L_IoU_* to *L_α_*_-*IoU*_, we found that α-IoU loss could adapt the loss value of all objects. The same was true for CIoU and α-CIoU loss. Thus, we took the IoU and α-IoU loss functions as examples, and the derivation proceeded as follows:

(5) $$\begin{array}{l} w_{L\tau }=L_{\alpha \text{-}IoU}/L_{IoU}=1+(IoU-IoU^{\alpha })/(1-IoU),\\ \Rightarrow w_{L\tau }(IoU=0)=1,\\ \Rightarrow \underset{IoU\to 1}{\lim}w_{L\tau }=\alpha . \end{array}$$

When 0 < *α* < 1, the reweighting factor *w_Lτ_* decreases with the decrease in *IoU*. When *α* > 1, the reweighting factor *w_Lτ_* increases monotonically with the increase in *IoU*; thus, α-CIoU loss could help the detector to focus on high-IoU objects, which means a greater focus on high-quality detection boxes with minor regression errors.

The number of high-quality anchor boxes with minor regression errors in object detection is generally much lower than the number of low-quality anchor boxes, and low-quality objects can produce excessive gradients that affect the training process [41]. Therefore, we increased the loss weight of high-IoU objects by controlling *α* > 1, which could significantly improve the training performance in the late stages and further improve the accuracy of model localization and detection.

In summary, the introduction of α-CIoU could optimize the positive and negative object imbalance problem in the bounding box regression task. In other words, α-CIoU could reduce the optimization contribution of low-quality anchor boxes presenting less overlap with ground-truth boxes, allowing the regression process to focus on high-IoU objects. Ultimately, it was demonstrated that α-CIoU effectively improves the regression accuracy of the bounding box of YOLOv5s by adaptively reweighting the losses and gradients of objects without increasing the number of parameters and the training/inference time of the model.

## 4. Experiments

### 4.1. Experimental Setup

#### 4.1.1. Datasets and Evaluation Metrics

To confirm the effectiveness of our proposed model, we carried out experiments on two public aerial image benchmark datasets, DIOR [1] and VisDrone [42].

**Figure 6:**
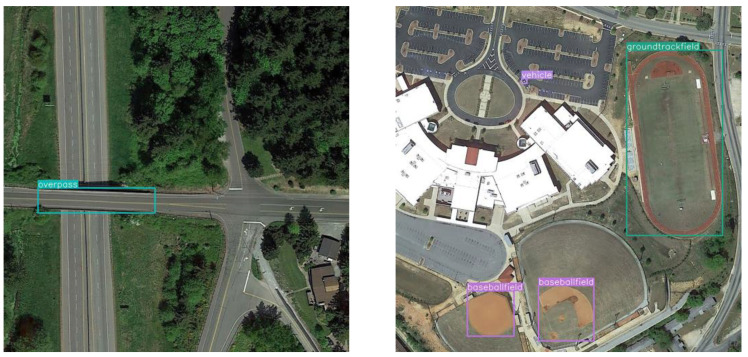
Sample images from the DIOR dataset.

The DIOR dataset is a large-scale, publicly available image dataset for optical remote-sensing image object detection containing 23,463 images and over 190,000 instances. The dataset covers 20 object classes, which include stadiums, dams, baseball fields, etc. The resolution of the images in the dataset is 800 × 800 pixels. The challenges associated with object detection in the DIOR dataset are multifaceted. First and foremost, the sheer volume of object classes, instances, and images presents a substantial task. Secondly, the objects within the dataset vary considerably in scale, leading to significant disparities in the imaging results. Thirdly, the objects to be detected exhibit a high degree of inter-class similarity and intra-class diversity, further complicating the detection process. Figure 6 provides a visual representation of these challenges, showcasing several images for object detection within the DIOR dataset.

**Figure 7:**
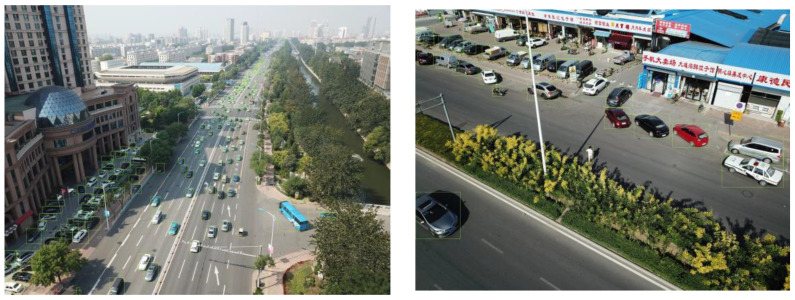
Sample images from the VisDrone dataset.

The VisDrone dataset is a UAV-based visual dataset of optical aerial images. The VisDrone dataset encompasses 10,209 static images, captured through a variety of UAV-mounted cameras, ensuring extensive coverage. The dataset includes 10 distinct object classes, such as pedestrians, buses, and trucks. Impressively, each object class averages over 50,000 instances, contributing to the comprehensive nature of this dataset. The resolution of the images in the dataset is as high as 2000 × 1500 pixels. The complexities of object detection in the VisDrone dataset are as follows: Firstly, the dataset presents a vast array of detection challenges. Secondly, the distribution of these detection objects is not uniform, adding another layer of difficulty. Thirdly, many of these objects are heavily obscured, further complicating their identification and detection. Figure 7 provides visual examples of these challenges, illustrating several instances of object detection within the VisDrone dataset.

According to the definition of the absolute size of objects in MS COCO [8], a common dataset in the field of object detection, small objects comprise less than 32 × 32 pixels, medium objects between 32 × 32 and 96 × 96 pixels, and large objects more than 96 × 96 pixels. As shown in Table 1, on the one hand, the VisDrone dataset and the DIOR dataset differ in the number of large and small objects. On the other hand, they could not allow the baseline model to effectively detect small objects. Therefore, using these datasets to verify the advantages of the proposed model for small-object detection in aerial images was reasonable.

We used the criteria proposed by Microsoft in the publicly available image dataset MS COCO to evaluate the performance of the object detectors [8]. We selected six main metrics to measure the performance of the proposed model, namely *AP*_50_, *AP*_75_, *AP*_50:95_, *AP_S_*, *AP_M_*, and *AP_L_*. *AP*_50_ and *AP*_75_ denote average precision (AP) values corresponding to an IoU of 0.5 and 0.75, respectively. *AP*_50:95_ is a primary challenge metric relative to the previous two metrics. It represents the mean AP value under an IoU from 0.5 to 0.95 in steps of 0.05. *AP_S_*, *AP_M_*, and *AP_L_* correspond to the mean AP values for small, medium, and large objects, respectively. Since we focused on improving the detection of small objects in aerial images, *AP_S_* was used as the main metric for the experiments. To evaluate the performance of the model more comprehensively in small-object detection, we introduce some important metrics such as *P* (precision), *R* (recall), and the F1 score to measure the model precision, recall, and the summed mean of the model precision and recall. We also selected the inference time and parameters to evaluate the detection speed and size of the model comprehensively.

#### 4.1.2. Implementation Details

Our proposed EL-YOLOv5 model was implemented on PyTorch, and the training and testing of all variant models were completed on an NVIDIA GeForce RTX 3080Ti GPU with 12 GB memory. In all experiments, we trained the VisDrone and DIOR datasets for 200 epochs with a batch size of 8. We set the initial learning rate to 0.01 and dynamically decreased it using a cosine annealing decay strategy. Before the images are input into the model, we performed a unified pre-processing operation on the images within two datasets to unify the image size to 640 × 640 pixels and then input to the model for feature extraction and the subsequent training process. In addition, we used the unified default data-augmentation strategies and default parameters of the YOLO detector for all experiments.

According to the needs of realistic scenarios, we chose YOLOv5s as the baseline model in the experiments. Furthermore, to verify the robustness of the proposed modifications model, we transferred all the modifications made on S to other scales and compared the experimental results for analysis.

Other detection models, such as the Scaled-You Only Look Once version 4 (Scaled-YOLOv4) [43], TPH-YOLOv5, and YOLOv7 were validated using the default settings from the relevant literature.

### 4.2. Experimental Results

#### 4.2.1. Experimental Results of the Model Architecture

We conducted comparative experiments on the baseline model and three models with different modified architectures using DIOR and VisDrone datasets, considering five metrics (*AP*_50_, *AP*_75_, *AP*_50:95_, *AP_S_*, and parameters). The results are shown in Table 2 and Table 3.

By comparing the baseline model and Models 1, 2, and 3, as presented in Figure 3, it can be observed that all of the modified models have improved their *AP_S_* by approximately 2% compared to the baseline model. Therefore, introducing low-level feature maps in YOLOv5s effectively enhanced the detection accuracy of the model for small objects. The comparison between Model 1 and Models 2 and 3 showed that Model 1 had slightly more parameters than Models 2 and 3 but significantly outperformed them in terms of *AP*_50:95_. Our analysis suggested that although reducing deep feature maps and detection heads for large objects reduced the computational resources, it also reduced the depth and complexity of the model, which indirectly impacted its performance. It also affected the detection accuracy for small objects when the detector complexity was overly low.

**Figure 8:**
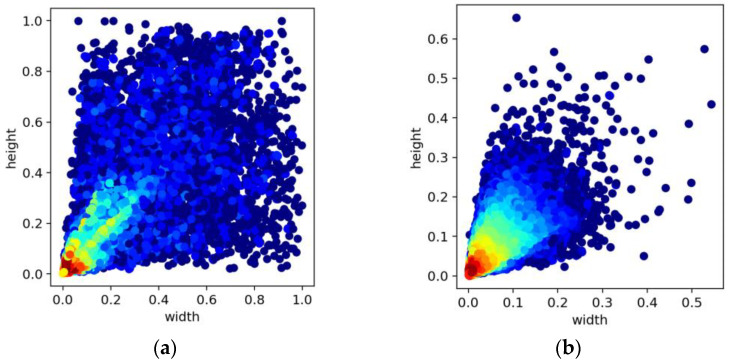
(**a**) Distribution of objects with different widths and heights in the DIOR dataset; (**b**) distribution of objects with different widths and heights in the VisDrone dataset.

A comparison of Table 2 and Table 3 reveals that for the UAV-based VisDrone dataset, the introduction of low-level feature maps resulted in a more significant improvement in model performance. On the contrary, for the remote-sensing DIOR dataset, although the modified models presented an improved *AP_S_* compared to the baseline model, the *AP*_50:95_ of Models 1, 2, and 3 all decreased to different degrees. We hypothesized that two primary factors led to this phenomenon. On the one hand, as illustrated in Figure 8a,b, the distribution of small, medium, and large objects in the DIOR dataset was more balanced, whereas, in the VisDrone dataset, small objects dominated. Our modified models could detect a larger quantity of small objects. However, from Figure 6, we noted that many small objects in the DIOR dataset were not annotated. As a result, the *AP*_50:95_ in the DIOR dataset decreased. In contrast, in the unique context of the VisDrone dataset, the model’s average precision was significantly enhanced. On the other hand, the introduction of high-resolution feature maps provided more detailed information in favor of small objects, but it also introduced background noise, which was detrimental to the model’s performance.

Through multiple rounds of experiments, it was clear that Model 1 outperformed the baseline model in detecting small objects across both datasets. Therefore, after considering the algorithm models’ average accuracy, complexity, and number of model parameters, we ultimately chose the network architecture of Model 1.

#### 4.2.2. Experimental Results of ESPP

To demonstrate the benefits of our ESPP design for aerial image object detection, we selected a series of currently popular spatial pyramid pooling modules for comparison experiments with ESPP. All included spatial pyramid pooling modules could directly replace the original SPP method, including SPPF [15], simplified SPPF (SimSPPF) [16], SPPCSPC [17], atrous spatial pyramid pooling (ASPP) [44], RFB, and ESPP.

We ensured that all hyper-parameters and configurations remained the same and replaced SPP with each of the abovementioned modules on top of the baseline YOLOv5s model. We conducted experiments on the DIOR and VisDrone datasets separately, comparing the five metrics *AP*_50_, *AP*_75_, *AP*_50:95_, *AP_S_*, and the number of parameters. The results for the impact of the different SPP modules on the performance of YOLOv5s on the DIOR and VisDrone datasets are shown in Table 4 and Table 5.

As observed in Table 4 and Table 5, ESPP performed significantly better than the other modules on the two aerial image datasets. For the DIOR dataset, ESPP improved the *AP*_50:95_ by 0.7% and the *AP_S_* by 0.8% compared to SPP. For the VisDrone dataset, ESPP improved the *AP*_50:95_ by 1.2% and the *AP_S_* by 0.7% compared to SPP. Interestingly, ESPP is a lightweight module that exhibited only a minor increase in the number of parameters.

The improved accuracy observed in our analysis was due to two main reasons. Firstly, ESPP improved the representational power of the feature maps through a serial convolution plus parallel convolution architecture, which enabled the fusion of local and global information at the spatial level and improved the average accuracy of the model. Secondly, ESPP enriched the feature maps with contextual information by introducing atrous convolution to increase the receptive fields, which was beneficial to small objects. In summary, ESPP is suitable for application in the network architecture of aerial image small-object detectors.

#### 4.2.3. Experimental Results of EL-YOLOv5

To verify the robustness of the proposed modified model, we transferred all of the modifications made on S to other scales and compared the experimental results for analysis. The experimental results are shown in Table 6 and Table 7.

Table 6 and Table 7 show that EL-YOLOv5s enhanced the *AP_S_* by 1.9% and 2.2% on two challenging aerial image datasets compared with the YOLOv5s. Thus, it was clear that our proposed model effectively solved the original YOLOv5 model’s problem of low accuracy in detecting small objects in aerial images. Although EL-YOLOv5 showed a slight increase in the inference time compared to the baseline model, the S-scale EL-YOLOv5 model achieved the requirement of real-time processing while maintaining a high level of detection accuracy. In addition, regarding the number of parameters, the chip of the UAV and processor required less than 10 MB for the model in most cases, and our EL-YOLOv5s fully met the requirements of embedded deployment. Therefore, our EL-YOLOv5s can run on most UAV processors. Meanwhile, the optimization effect of EL-YOLOv5 was more visible on the VisDrone dataset, probably originating from the denser distribution of small objects in this dataset.

By comparing the *AP_S_*, *AP_M_*, and *AP_L_* in Table 6 and Table 7, we further analyzed the scale problem of the objects in the two datasets. On the one hand, based on Table 1, we found that low-scale objects dominated in the VisDrone dataset. Therefore, the *AP*_50:95_ was effectively improved when our experiments were optimized for small objects, and correspondingly the *AP_L_* registered an approximately 8% growth. Meanwhile, it can be found that the improvement in detection accuracy of large-scale objects was much higher than that of low-scale objects, which also reflected the difficulty of improving the accuracy of small objects from the side. On the other hand, we found that the *AP_M_* and *AP_L_* both decreased slightly in the DIOR dataset. Our analysis suggested that the proportion of large-scale objects was close to that of small objects in the DIOR; therefore, when the experimental model architecture highlights the optimization of the accuracy for small objects, the *AP_M_* and *AP_L_* would be affected accordingly. Then, for the detection task of large-scale objects in the satellite remote-sensing object detection scenario, our proposed EL-YOLOv5 exhibits some limitations.

To demonstrate the detection performance of EL-YOLOv5 for different categories of objects, we selected the more challenging aerial image dataset VisDrone in small-object detection and the S-scale YOLOv5 model. Then, we conducted experiments on different categories of objects in this dataset to obtain relevant metrics such as *P* and *R*, and the final experimental results are shown in Table 8. The arrows in Table 8 reflect the rising state of the data percentiles.

Our proposed algorithm exhibited different degrees of improvement for different categories of objects. Our analysis suggested that firstly, modifying the model architecture can make the model retain more detailed information favorable to small objects; secondly, the multibranch structure of ESPP can effectively enhance the feature-extraction ability of the model for small objects. And finally, the α-CIoU loss function can effectively alleviate the problem of positive and negative sample imbalance, and all three different improvements significantly increased the detection accuracy of different categories of objects in the dataset. Since our EL-YOLOv5 achieved an increase in points in all categories, this also reflected the model’s generalizability from this angle.

**Figure 9:**
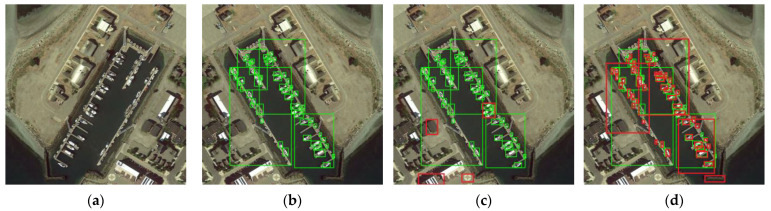
Qualitative results comparison between YOLOv5 and EL-YOLOv5 for the DIOR dataset. Ground truth and prediction are marked by green and red boxes, respectively. (**a**) An original image from the DIOR dataset; (**b**) the original image with ground truth box; (**c**) the original image is detected by YOLOv5; (**d**) the original image is detected by EL-YOLOv5.

**Figure 10:**
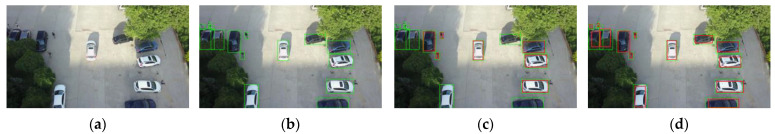
Qualitative results comparison between YOLOv5 and EL-YOLOv5 for the VisDrone dataset. Ground truth and prediction are marked by green and red boxes, respectively. (**a**) An original image from the VisDrone dataset; (**b**) the original image with ground truth box; (**c**) the original image is detected by YOLOv5; (**d**) the original image is detected by EL-YOLOv5.

Figure 9 and Figure 10 illustrated the qualitative results comparison between YOLOv5 and EL-YOLOv5 for the two datasets. By looking at Figure 9c,d and Figure 10c,d, we found that EL-YOLOv5 could effectively alleviate the low-scale object missing problem in the two aerial image datasets. In conclusion, it was highly intuitive that EL-YOLOv5 had a great advantage in small-object detection compared to the baseline model.

### 4.3. Ablation Experiments

To validate the effectiveness of the modules presented in this paper, we performed ablation experiments on the DIOR and VisDrone datasets. The experimental results are shown in Table 9 and Table 10.

By looking at Table 9 and Table 10, we can see that, firstly, the modification of the model architecture improved the accuracy of small-object detection more noticeably compared to the modification of ESPP and the loss function. Secondly, our proposed EL-YOLOv5s model achieved improvements of 1.9% and 2.2% in the *AP_S_* compared to YOLOv5s for both datasets, which illustrated that EL-YOLOv5 could indeed address the problem of low accuracy for small-object detection in aerial images. Thirdly, for the DIOR dataset, all our modules improved the small-object detection accuracy, but the noise introduced by the low-level feature maps when modifying the model architecture also affected the average accuracy of the model to a certain extent. Fourthly, EL-YOLOv5s fully satisfied the requirement of the number of parameters that could be embedded in the model for a realistic application scenario.

Delving deeper into the table data, it becomes evident that for the VisDrone dataset, a significant improvement in the *AP*_50:95_ was obtained by EL-YOLOv5s—an increase of 3.5% when compared with the baseline model. In contrast, for the DIOR dataset, enhancements merely to the model’s ESPP module and loss function led to a 1.1-point increase in the *AP*_50:95_ index. However, further adjustments to the model architecture in the DIOR dataset resulted in a decrease in the *AP*_50:95_ by 1.6%. This may have been due to the difference in the distribution of large and small objects between the two datasets. Therefore, for aerial image datasets that are not dominated by small objects, it would be more beneficial to enhance the feature representation of the model by improving the module in scenarios that are more demanding for average model accuracy.

### 4.4. Comparisons with Other Sota Detectors

The EL-YOLOv5 model was compared with other SOTA detectors on the DIOR and VisDrone datasets to prove the effective performance of the detectors to which our method was applied. These detectors included Scaled-YOLOv4, TPH-YOLOv5, YOLOv5, and YOLOv7.

**Figure 11:**
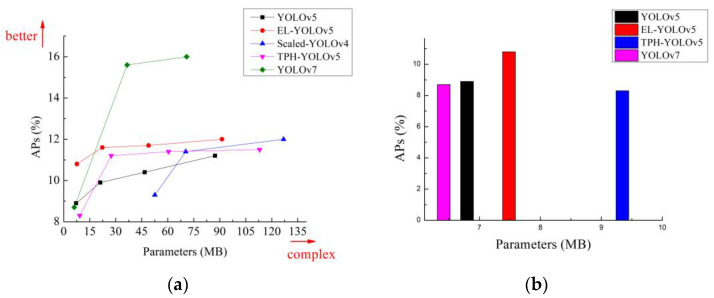
(**a**) Comparative analysis of small-object detection accuracy across different detectors using the DIOR dataset; (**b**) the small-object detection accuracy within the DIOR dataset for different detectors with parameter sizes of less than 10 MB.

**Figure 12:**
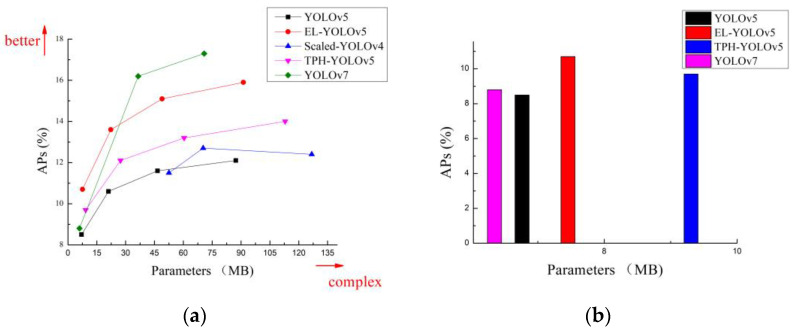
(**a**) Comparative analysis of small-object detection accuracy across different detectors using the VisDrone dataset; (**b**) the small-object detection accuracy within the VisDrone dataset for different detectors with parameter sizes of less than 10 MB.

Figure 11a and Figure 12a show that the detection accuracy of our proposed EL-YOLOv5 model for small objects in both datasets was significantly higher than that of advanced object detectors such as Scaled-YOLOv4, YOLOv5, and TPH-YOLOv5. Regarding the comparison between EL-YOLOv5 and YOLOv7, EL-YOLOv5 was more dominant on the S-scale. While EL-YOLOv5 was somewhat less accurate than YOLOv7 as the number of parameters increased, the extensive parameter number did not align with the requirements of embedded deployment. That is, when it comes to deployment within an embedded environment, the EL-YOLOv5 model exhibited significant advantages over YOLOv7.

For object-detection tasks in aerial imagery, we must consider the deployment requirements of the application area and further control the number of parameters in detector models. We mentioned near the beginning of this paper that FPGA chips [18] are generally used to implement cutting-edge technologies such as image processing and object detection. FPGAs typically possess less than 10 MB of on-chip memory and are devoid of any off-chip memory or storage. Therefore, lightweight models are more suitable for implementation using FPGAs, which are not constrained by the width of the storage bandwidth, while video frames can be processed in real time by FPGAs. Figure 11b and Figure 12b show that our proposed EL-YOLOv5 model performed optimally in terms of detection accuracy for small objects when the control model parameter count was less than 10 MB.

## 5. Conclusions

In this paper, we proposed EL-YOLO, a modified object-detection model applicable to the corresponding YOLO models, using S-scale YOLOv5 as the baseline model to address the problem of low accuracy for small-object detection in aerial images. First, we adapted three model architectures and validated them through multiple rounds of experiments, analyzing the reasons for their performance and architectural features and selecting the best-performing model. The best-performing model was proven to be able to maximize the accuracy in detecting small objects at a low cost in terms of computational resources. Second, we designed a novel ESPP method based on a human visual perception system to replace the original SPP module to further enhance the feature-extraction capability of the model for small targets. Finally, we introduced the α-CIoU loss function to optimize the positive and negative sample imbalance problem in the bounding box regression task, making it easier for the model to pinpoint small objects. Several rounds of experimental validation for our proposed EL-YOLOv5 model on the DIOR and VisDrone datasets were conducted, and it was finally demonstrated that the embeddable S-scale EL-YOLOv5 model achieved an *APs* of 10.8% on the DIOR dataset and 10.7% on the VisDrone dataset, which represented the highest accuracy among the existing lightweight model results.

Our proposed EL-YOLO model can be further optimized. For datasets where the proportion of small objects is not dominant, such as DIOR, we can further design new feature-enhancement modules to improve the feature-extraction capability of the model for small objects without modifying the model architecture. Our algorithm can also be combined with edge computing techniques, which can be used to process massive requirements near the data sources, ultimately enabling real-time decisions in real-world scenarios. In addition, other techniques can be well-suited to solving the aerial image object-detection problems that were not considered in this study, representing directions for future research.

## Figures and Tables

**Table 1:** The absolute pixel size distribution of the VisDrone and DIOR training sets.

Dataset	<32^2^ Pixels	32^2^–96^2^ Pixels	>96^2^ Pixels
VisDrone	164,627	94,124	16,241
DIOR	12,792	7972	5966

**Table 2:** Experiment results using different model architectures for the DIOR dataset.

Method	*AP*_50_ (%)	*AP*_75_ (%)	*AP*_50:95_ (%)	*AP_S_* (%)	Parameters (M)
Baseline Model	79.4	61.8	57.1	8.9	7.11
Model 1	78.7	59.0	**54.7**	**11.1**	7.25
Model 2	77.5	58.4	53.9	10.5	5.44
Model 3	65.2	44.4	42.3	8.7	1.77

**Table 3:** Experiment results using different model architectures for the VisDrone dataset.

Method	*AP*_50_ (%)	*AP*_75_ (%)	*AP*_50:95_ (%)	*AP_S_* (%)	Parameters (M)
Baseline Model	27.4	14.2	14.9	8.5	7.08
Model 1	31.6	17.2	**17.8**	**10.6**	7.22
Model 2	31.3	16.4	17.3	10.6	5.43
Model 3	30.9	15.5	16.6	10.3	1.75

**Table 4:** Experimental results using different SPP modules for the DIOR dataset.

Method	*AP*_50_ (%)	*AP*_75_ (%)	*AP*_50:95_ (%)	*AP_S_* (%)	Parameters (M)
YOLOv5s + SPP	79.4	61.8	57.1	8.9	7.11
YOLOv5s + SPPF	79.2	61.7	57.1	9.1	7.11
YOLOv5s + SimSPPF	79.4	61.7	57.1	8.8	7.11
YOLOv5s + SPPCSPC	79.1	61.4	56.6	8.7	10.04
YOLOv5s + ASPP	79.2	61.4	56.7	9.0	15.36
YOLOv5s + RFB	78.8	62.1	57.2	8.3	7.77
YOLOv5s + ESPP	79.6	62.9	**57.8**	**9.7**	7.44

**Table 5:** Experimental results using different SPP modules for the VisDrone dataset.

Method	*AP*_50_ (%)	*AP*_75_ (%)	*AP*_50:95_ (%)	*AP_S_* (%)	Parameters (M)
YOLOv5s + SPP	27.4	14.2	14.9	8.5	7.08
YOLOv5s + SPPF	27.2	14.2	14.9	8.5	7.08
YOLOv5s + SimSPPF	27.7	14.3	15.1	8.7	7.08
YOLOv5s + SPPCSPC	27.2	14.1	14.9	8.4	10.01
YOLOv5s + ASPP	27.1	14.1	14.9	8.3	15.34
YOLOv5s + RFB	27.1	14.4	15.0	8.5	7.74
YOLOv5s + ESPP	28.4	15.9	**16.1**	**9.2**	7.42

**Table 6:** Performance comparison of YOLOv5 and EL-YOLOv5 across various scales within the DIOR dataset.

Method	Scales	*P* (%)	*R* (%)	*AP*_50:95_ (%)	*AP_S_* (%)	*AP_M_* (%)	*AP_L_* (%)	F1 Score	Parameters (M)	Inference Time (MS)
YOLOv5	S	88.0	76.1	57.1	8.9	38.4	69.4	0.82	7.11	14.6
	M	88.8	77.6	60.0	9.9	39.0	72.7	0.83	21.11	22.1
	L	90.2	77.5	61.8	10.4	40.1	74.6	0.83	46.70	25.2
	X	90.3	79.1	63.1	11.2	41.8	76.5	0.84	87.33	29.7
	S	83.6	74.2	55.5	10.8	36.4	67.1	0.79	7.59	18.2
	M	84.8	76.8	58.5	11.6	38.2	70.7	0.81	22.31	26.7
EL-YOLOv5	L	85.8	77.4	60.5	11.7	40.6	72.8	0.81	49.04	30.9
	X	87.8	77.7	61.8	12.0	39.1	74.3	0.82	91.29	37.8

**Table 7:** Performance comparison of YOLOv5 and EL-YOLOv5 across various scales within the VisDrone dataset.

Method	Scales	*P* (%)	*R* (%)	*AP*_50:95_ (%)	*AP_S_* (%)	*AP_M_* (%)	*AP_L_* (%)	F1 Score	Parameters (M)	Inference Time (MS)
YOLOv5	S	46.8	36.1	14.9	8.5	22.4	30.2	0.41	7.08	19.7
	M	53.9	38.2	17.9	10.6	26.5	32.1	0.45	21.07	24.0
	L	55.4	39.9	19.4	11.6	28.6	40.7	0.46	46.65	28.1
	X	56.9	40.9	20.0	12.1	29.5	39.0	0.48	87.26	31.8
	S	50.9	39.7	18.4	10.7	27.1	37.9	0.45	7.56	34.0
	M	54.1	44.5	21.4	13.6	30.8	40.7	0.49	22.27	37.1
EL-YOLOv5	L	57.9	45.8	22.9	15.1	32.3	42.0	0.51	48.98	41.0
	X	56.0	48.4	23.7	15.9	33.2	44.7	0.52	91.21	47.3

**Table 8:** The detection performance of each category comparison of YOLOv5s and EL-YOLOv5s within the VisDrone dataset.

Category	YOLOv5s.			EL-YOLOv5s		
	*P* (%)	*R* (%)	*mAP*_50:95_ (%)	*P* (%)	*R* (%)	*mAP*_50:95_ (%)
pedestrian	50.7	39.3	15.4	61.6	41.1	18.7 ↑3.3
people	46.2	34.9	10.1	49.4	31.7	10.5 ↑0.4
bicycle	28.0	15.9	3.76	30.5	15.9	5.32 ↑1.56
car	63.7	74.2	47.1	74.4	79.1	53.6 ↑6.5
van	48.6	38.0	24.1	45.7	45.7	28.1 ↑4.0
truck	52.0	33.7	18.0	51.4	37.2	22.9 ↑4.9
tricycle	42.6	24.8	9.68	44.3	29.4	13.5 ↑3.82
awning-tricycle	26.9	13.5	5.74	25.6	22.2	8.36 ↑2.62
bus	60.2	42.2	26.1	51.4	53.1	37.6 ↑11.5
motor	49.2	38.7	14.7	42.9	44.4	18.0 ↑3.3

**Table 9:** The effects of different module combinations in YOLOv5s for the DIOR dataset.

Method	*P* (%)	*R* (%)	*AP*_50:95_ (%)	*AP_S_* (%)	*AP_M_* (%)	*AP_L_* (%)	F1 Score	Parameters (M)
YOLOv5s	88.0	76.1	57.1	8.9	38.4	69.4	0.82	7.11
YOLOv5s + Model 1	84.1	75.8	54.7	11.1	37.2	66.2	0.80	7.25
YOLOv5s + ESPP	88.9	76.1	57.8	9.7	37.4	70.3	0.82	7.44
YOLOv5s + α-CIOU	87.0	75.8	57.5	9.8	38.5	69.8	0.81	7.11
YOLOv5s + ESPP + α-CIOU	89.1	76.9	58.2	10.2	38.8	70.5	0.83	7.44
EL-YOLOv5s	83.6	74.2	55.5	10.8	36.4	67.1	0.79	7.59

**Table 10:** The effects of different module combinations in YOLOv5s for the VisDrone dataset.

Method	*P* (%)	*R* (%)	*AP*_50:95_ (%)	*AP_S_* (%)	*AP_M_* (%)	*AP_L_* (%)	F1 Score	Parameters (M)
YOLOv5s	46.8	36.1	14.9	8.5	22.4	30.2	0.41	7.08 M
YOLOv5s + Model 1	51.8	39.2	17.8	10.6	25.8	34.1	0.45	7.22 M
YOLOv5s + ESPP	46.8	36.9	16.1	9.2	24.5	34.4	0.41	7.42 M
YOLOv5s + α-CIOU	50.3	34.0	16.2	9.5	24.1	31.3	0.41	7.08 M
YOLOv5s + ESPP + α-CIOU	50.8	36.5	16.5	9.9	24.2	35.3	0.43	7.42 M
EL-YOLOv5s	50.9	39.7	18.4	10.7	27.1	37.9	0.45	7.56 M

## Data Availability

The datasets used in this study are all public datasets.
